# Outcomes of intravitreal dexamethasone implant (Ozurdex^®^) in patients with post-surgical macular edema – a real-world scenario

**DOI:** 10.1186/s40942-024-00626-5

**Published:** 2025-01-10

**Authors:** Elder Ohara de Oliveira Júnior, Isabel Ahn, Rodrigo Antonio Brant Fernandes, Arthur Gustavo Fernandes

**Affiliations:** 1 Ophthal – Hospital Especializado, São Paulo, SP Brazil; 2 Department of Ophthalmology and Visual Sciences, Paulista Medical School, Federal University of São Paulo – UNIFESP, São Paulo, SP Brazil; 3 Roski Eye Institute, University of Southern California, Los Angeles, CA USA; 4 Department of Anthropology and Archaeology, University of Calgary, 2500 University Dr NW, Calgary, AB T2N 1N4 Canada

**Keywords:** Treatment, Outcomes, Ozurdex, Macular edema

## Abstract

**Background:**

Clinically significant macular edema (CME) is the leading cause of visual loss after ophthalmologic surgery due to the release of inflammatory mediators promoted by the procedures. We aimed to evaluate the outcomes of intravitreal Ozurdex^®^ (700 µg dexamethasone) implants as a primary therapeutical option for post-surgical macular edema cases.

**Methods:**

Patients with post-surgical macular edema diagnosed by optical coherence tomography (Cirrus SD-OCT) and treated with Ozudex were selected for the current study. Data was retrospectively collected from medical records from January 2020 to December 2022 and included sex, age, laterality, and timeline of treatment (i.e. implant alone or at the time of silicon oil removal in cases requiring vitreorretinal surgery). Complications associated with treatment were also noted as well as the need of further treatments. The structural analysis focused on measuring central macular thickness (CMT—average thickness within the 1 mm circle of the ETDRS) from the internal limiting membrane to the Bruch’s membrane complex, as well as the average total macular thickness including parafoveal and perifoveal regions determined by the device (CAT). The functional evaluation was based on the best-corrected visual acuity (VA) measured in logMAR.

**Results:**

A total of 46 participants were included (56.2% males, mean age: 60.9 ± 11.2 years old). A statistically significant change was observed in the postoperative versus the preoperative period for all parameters (*p* < 0.05). The mean VA difference was − 0.17 ± 0.24; CMT was − 109.22 ± 124.85 and CAT was − 14.76 ± 58.95. We observed a significant effect of the moment of Ozurdex implantation on VA improvement, so that cases with implantation at the time of oil removal showed lower improvement than cases with implantation at a distinct timing (Coef. 0.19, 95%CI: 0.02 to 0.36, *p* = 0.027). Eleven cases (23.9%) required further treatment such as new Ozurdex implantation (8 cases) or surgery (3 cases). Only one case (2.17%) showed increased intraocular pressure and underwent glaucoma surgery.

**Conclusions:**

Intravitreal Ozurdex implants significantly improved functional and structural aspects in post-surgical macular edema. The timing of implantation influenced VA improvement, with a distinct step approach showing better outcomes than at the time of oil removal.

## Introduction

Post-surgical macular edema, specifically following cataract surgery (Irvine-Gass Syndrome), was first described by Irvine in 1953 [1]. Its pathogenesis was later elucidated by Gass and Norton based on fluorescein angiography [2]. Although the exact cause remains uncertain, the increase in inflammatory substances such as prostaglandin E2 and cytokines like IL-1b and CCL2 has been postulated as a contributing factor [3].

The incidence of Irvine-Gass Syndrome ranges from 0.1 to 2.4% for clinically significant macular edema and from 4.0 to 11% when assessed using optical coherence tomography (OCT) [4, 5]. Studies have shown an incidence of 16.3% following pars plana vitrectomy for the treatment of rhegmatogenous retinal detachment, with a higher risk in cases involving the macula, duration longer than one week, and the presence of proliferative vitreoretinopathy (PVR) [6]. With the potential for permanent visual damage, this complication remains one without evidence-based therapies according to current guidelines.

The slow-release Dexamethasone implant (Ozurdex) is an on-label therapy for the treatment of non-infectious posterior uveitis, providing sustained release in the vitreous for up to six months. Due to its inhibitory effect on prostaglandins and cytokines possibly related to the pathogenesis of macular edema, cases of post-surgical macular edema could be benefit by the treatment with Ozurdex, aiming improvement on visual acuity and decrease of macular thickness observed through OCT. The literature, however, does not show a consensus among the ophthalmological society on regards of the treatment outcomes [4, 5, 7–12].

The purpose of the current study was to evaluate the outcomes of intravitreal Ozurdex^®^ (700 µg dexamethasone) implantation in cases of post-surgical macular edema, showing its effectiveness and safety on improving both structural (macular thickness) and functional (visual acuity) parameters.

## Methods

A retrospective study was carried out by medical chart review of patients diagnosed with post-surgical macular edema and treated with Ozurdex at the Retina Division from the Ophthal Hospital Especializado Ltda, Sao Paulo, Brazil, in the period of January 2020 to December 2022. This study was approved by the HOlhos Institutional Review Boards and was carried out in accordance with the tenets of the Declaration of Helsinki.

We selected patients diagnosed with post-surgical macular edema confirmed by OCT, who underwent treatment with Ozurdex implant, and were followed up for a continuous period of 6 months. All OCT images were acquired using the ZEISS Cirrus HD-OCT Model 4000 (Carl Zeiss-Meditec, Dublin, CA). Data on sex, age, laterality, and treatment timeline (i.e. Ozurdex^®^ implant alone or concurrent Ozurdex^®^ implant with silicone oil removal) were recorded. Ozurdex^®^ implant alone refers to cases where the Ozurdex^®^ implant was administered independently, during a standalone procedure, without any simultaneous surgical interventions while concurrent Ozurdex^®^ implant with silicone oil removal refers to cases where the Ozurdex^®^ implant was administered immediately after the removal of silicone oil during the same surgical session. Complications associated with treatment and the need for further treatment were assessed.

Exclusion criteria were applied to ensure a homogeneous study population and to minimize potential confounding factors. Patients with diabetic retinopathy or retinal vascular occlusion were excluded because these conditions are independent causes of macular edema with distinct pathophysiologies that could confound the treatment outcomes. Patients with an epiretinal membrane (ERM) causing macular distortion were excluded as such structural changes could interfere with accurate assessment of macular edema and visual outcomes. ERM tractional effect may be associated with worsening visual acuity or metamorphopsia, so exclusion of these patients was important to eliminate the confounding effect of pre-existing vision-related symptoms unrelated to post-surgical macular edema treatable with Ozurdex. Additional exclusions included patients with a history of acute coronary or cerebrovascular events in the past 12 months due to the potential systemic risks associated with corticosteroid therapy, pregnant individuals due to contraindications and ethical considerations related to treatment safety during pregnancy, and patients undergoing secondary intraocular lens implantation with scleral fixation as it is a formal contraindication of Ozurdex^®^ implantation.

The structural analysis focused on measuring central macular thickness (CMT—average thickness within the 1 mm circle of the ETDRS) from the internal limiting membrane to the Bruch’s membrane complex, as well as the average total macular thickness including parafoveal and perifoveal regions determined by the device (CAT), with particular attention to the proper positioning and segmentation of the retinal layers. The functional evaluation was based on the best-corrected visual acuity (VA) measured in logMAR.

Statistical analyzes were performed using Stata/SE Statistical Software, Release 14.0, 2015 (Stata Corp, College Station, Texas, USA). Changes in VA, CMT, and CAT from baseline to post-Ozurdex injection were investigated using Wilcoxon rank-sum test. The relationships between improvements in outcomes were assessed using Spearman’s rank correlation coefficient. Factors associated with the magnitude of changes in VA, CMT, and CAT were analyzed using multiple linear regression models. Independent variables included sex, age, treatment timeline (implant alone or at the time of silicone oil removal), and baseline values for each outcome measure. These variables were selected based on their clinical relevance and potential influence on treatment outcomes. For all tests, a p-value < 0.05 was considered statistically significant.

## Results

A total of 46 eyes of 46 patients were selected to the current study. Table 1 shows the cases’ characteristics.

**Table 1:** Cases descriptive characteristics

Sex *N*(%)	
Male Female	20 (43.48) 26 (56.52)
Age *mean ± std*	60.91 ± 11.23 (60.00)
Laterality *N* (%)	
OD OS	24 (52.11) 22 (47.83)
Timing of implant *N* (%)	
During oil removal Independent	18 (39.13) 28 (60.87)

Table 2 shows the comparison of visual acuity, central macular thickness, and average total macular thickness pre- and post-treatment.

**Table 2:** Comparison of outcomes pre- and post-treatment

Criteria	Pre-treatment	Post-treatment	*p*-value
	mean ± sd (median)	mean ± sd (median)	
VA (logMAR)	0.41$\:\pm\:$0.24 (0.40)	0.22 $\:\pm\:0.16\:\left(0.25\right)$	< 0.0001
CMT	431$\:\pm\:$100.41 (484.00)	322.43$\:\pm\:$97.85 (306.50)	< 0.0001
CAT	316$\:\pm\:$50.57 (310.50)	299.11$\:\pm\:$39.29 (292.00)	0.0082

A statistically significant change was observed in the post-treatment period when compared to the baseline for all parameters (*p* < 0.05). The mean difference in VA was − 0.17 ± 0.24 (-0.125) logMAR; in CMT it was − 109.22 ± 124.85 (-105.50) µm; and in CAT it was − 14.76 ± 58.95 (-22.50) µm. There was a significant correlation between the functional (AV) and structural (CMT) improvements (rho = 0.4252, *p* = 0.0109).

Table 3 shows the results from the multiple linear regressions applied to investigate factor associated to the magnitude of change in visual acuity, central macular thickness, and average total macular thickness pre- and post-treatment.

**Table 3:** Multiple linear regressions to investigate factors associated with the outcomes, adjusted for sex, age, timing of implant and pre-treatment parameters

	Visual acuity		Central macular thickness		Average total macular thickness	
	Coefficient (95% CI)	*p*-value	Coefficient (95% CI)	*p*-value	Coefficient (95% CI)	*p*-value
Sex						
Female Male	Reference 0.05 (-0.12 to 0.22)	--- 0.586	Reference 21.96 (-39.88 to 83.80)	--- 0.486	Reference -5.26 (-29.64 to 19.12)	--- 0.672
Age	-0.01 (-0.01 to 0.01)	0.551	0.70 (-1.94 to 3.34)	0.604	-0.82 (-1.87 to 0.23)	0.125
Timing						
Independent Oil removal	Reference 0.19 (0.02 to 0.36)	--- **0.027**	Reference 21.93 (-44.46 to 88.31)	--- 0.517	Reference 18.99 (-5.72 to 43.72)	--- 0.132
Baseline measurement	-0.44 (-0.90 to 0.02)	0.059	-0.73 (-1.04 to -0.42)	**< 0.001**	-0.88 (-1.12 to -0.64)	**< 0.001**

The results indicate that sex, age, or pre-treatment visual acuity did not have a significant effect on the differences observed between pre- and post-treatment visual acuity (*p* < 0.05). However, the timing of the Ozurdex implant did show an effect, with cases where the implant was placed at the time of oil removal showing less improvement. Specifically, cases where the implant was done at the time of oil removal had 0.19 logMAR units less improvement in visual acuity compared to cases where the implant was placed at an independent time (Coef.: 0.19; 95% CI: 0.02 to 0.36; *p* = 0.027).

In terms of structure, sex, age, or timing of the implant did not have a significant effect on the differences observed between pre- and post-treatment CMT or CTA (*p* < 0.05). However, a statistically significant effect of the baseline measurements was observed on the differences pre- and post-treatments, such that the greater the baseline thickness, the larger the difference observed after treatment. For each 1 μm increase at baseline, the difference after treatment on CMT increases by 0.73 μm (Coef.: -0.73; 95% CI: -1.04 to -0.42; *p* < 0.001) and in CTA increases by 0.88 μm (Coef.: -0.87; 95%CI: -1.12 a -0.64; *p* < 0.001).

**Fig. 1:**
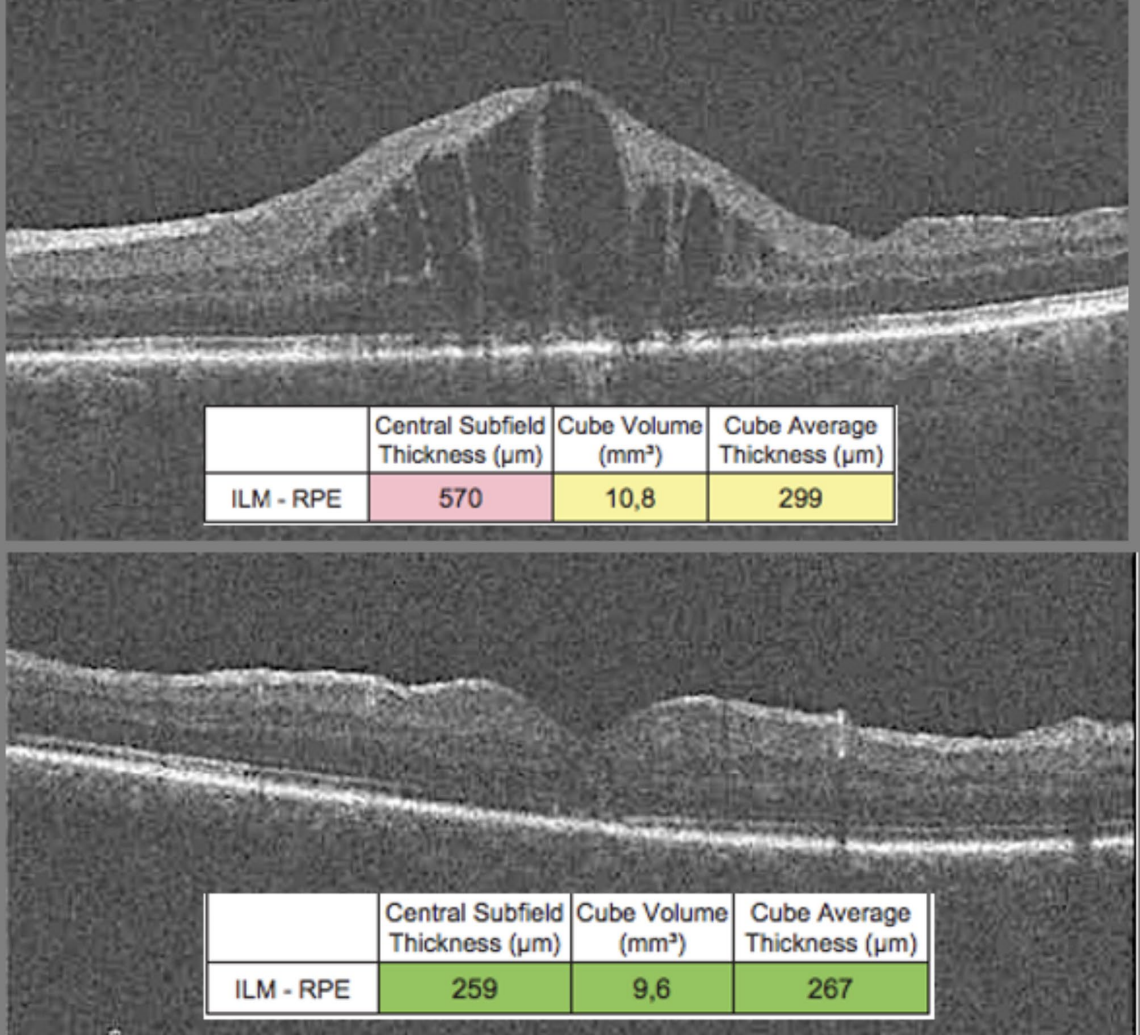
Anatomical differences observed on Cirrus-SD OCT pre- and post- Ozurdex implantation. A regression of intraretinal cysts and a decrease in macular thickness values are observed. It is important to note the loss of subfoveal outer retinal integrity as an anatomical sequela of chronic retinal damage

Figure 1 illustrates a patient who showed satisfactory structural improvement of the macula evaluated by OCT, three months after intravitreal Ozurdex treatment.

Of the 46 eyes evaluated, 11 (23.91%) required further treatment. Among the retreated patients, 7 (15.22%) needed an additional Ozurdex application due to partial functional and structural response. Four patients (8.70%) were referred for vitrectomy combined with peeling due to the development of an epiretinal membrane with a tangential traction component. One patient experienced a persistent increase in intraocular pressure, refractory to medical therapy (maintaining IOP at 32 mmHg despite hypotensive agents), and underwent trabeculectomy surgery.

## Discussion

The study conducted in a real-life setting shows functional and anatomical improvement after treatment of postoperative macular edema with an Ozurdex^®^ intravitreal implant. With a 6-month follow-up, an average improvement in visual acuity (VA) from 0.40 to 0.22 logMAR, in central macular thickness (CMT) from 431 to 322 μm, and in central area thickness (CAT) from 316 to 299 μm was observed. Moreover, our results also indicate that greater structural improvement in CMT is associated with better functional recovery in VA.

Among the risk factors for cystoid macular edema after rhegmatogenous retinal detachment, complex retinal detachment repairs that require multiple surgeries and pseudophakic or aphakic status are prominent [13]. The literature presents conflicting results, with some studies showing no anatomical improvement [13] and a recent publication finding significant differences in best corrected visual acuity and central macular thickness before and after the Ozurdex^®^ injection, with at least an 18-month follow-up [14]. Pignatelli et al. demonstrated that Ozurdex implantation had a statistically significant effect at the time of silicone oil removal in 24 eyes previously undergoing vitreoretinal surgery for the treatment of rhegmatogenous retinal detachment, showing a reduction in mean macular thickness and improved visual acuity after 6 months. Favorable results and an acceptable safety profile were achieved, with intraocular pressure (IOP) elevation in only one eye, which was managed clinically. Macular status was significantly associated with visual acuity after Dexamethasone implantation [15]. It should be noted that the intraoperative implant must be performed safely when the eye is filled with balanced salt solution (BSS) to avoid retinal trauma due to the kinetic energy of the implant [16].

Other corticosteroids, such as Triamcinolone, also show benefits in terms of visual acuity improvement and anatomical macular changes [17]; however, they have a less favorable safety profile, with a higher risk of elevated IOP, and should not be used as an alternative treatment in randomized studies [18]. Furthermore, the use of Ozurdex is associated with a lower need for retreatments, as recently demonstrated in cases of Irvine-Gass syndrome refractory to topical therapy with nonsteroidal anti-inflammatory drugs and corticosteroids [19, 20], as well as in vitrectomized eyes [21], with functional improvement through microperimetry already documented [22].

We found no significant effect of sex or age on the differences observed between pre- and postoperative parameters. Preoperative CMT and CAT values have shown a significant impact in the outcomes, such that the worse the baseline parameter, the greater the observed improvement. The timing of the implant was also significantly associated with the functional improvement, with cases where the implant was placed at the time of oil removal showing less improvement. It is important to emphasize that patients with silicone oil tamponade presented with severe retinal detachment, with surgical indication due to significant functional and/or anatomical impairment, especially in the presence of macular involvement and vitreoretinal proliferation. Some of these patients underwent complex procedures and re-operations, which negatively affect the final visual prognosis due to irreversible damage to the photoreceptors, despite the improvement in macular edema after surgery. In that sense, the observed finding on functional improvement associated with time of implant can be confounded by the case severity and have to be interpreted with caution.

Approximately 25% of patients required additional treatment, with two-thirds of them needing an additional injection of Ozurdex. The need for a new implant primarily occurred due to the absence of a complete anatomical response after 90 days of treatment, although there was a decrease in macular thickness and intraretinal cysts compared to baseline. Surgical intervention was indicated in four cases due to the development of an epiretinal membrane with a tangential traction component, causing anatomical distortion and macular thickening. This progression can be attributed to the inflammatory component intrinsic to surgical trauma, leading to fibrocellular proliferation along the inner limiting membrane.

Real-life scenarios provide important insights but also illustrate the challenges of maintaining regular follow-up. Patient attrition posed a significant challenge in this study, reflecting real-world constraints in delivering care to a diverse patient population. Among the 82 eyes pre-selected for the study, 33 patients missed scheduled follow-ups due to treatment abandonment or delays. This was often attributed to logistical barriers such as long travel distances from rural areas to the reference center in São Paulo, compounded by socioeconomic constraints and cultural factors influencing health-seeking behaviors. Despite clear guidance, adherence to follow-up schedules remained difficult for some patients, emphasizing the need for tailored interventions. Additionally, attrition included losses due to clinical complications. One patient experienced implant migration to the anterior chamber, one had retinal detachment, and another developed a myopic neovascular membrane, leading to treatment cessation. These cases highlight the challenges associated with managing severe underlying eye conditions and the inherent risks of intravitreal implants. To address attrition in future studies, we recommend strategies such as providing transportation support, enhancing patient education on the importance of follow-ups, and using telemedicine for interim evaluations to reduce the burden of in-person visits. Furthermore, employing reminders through digital platforms or community health workers may improve adherence. To evaluate the potential impact of attrition on our findings, we conducted sensitivity analyses. By comparing baseline characteristics of patients who completed follow-ups with those lost to attrition, we found no significant differences in demographics or baseline clinical parameters, suggesting minimal bias introduced by attrition. However, we acknowledge that the reduced sample size may have limited the generalizability of our findings. In that sense, controlled clinical trials with comprehensive follow-up mechanisms remain crucial for producing more robust evidence.

The retrospective design of the study imposes further limitations. Although confounding factors were minimized by considering the study’s exclusion criteria, it is known that more complex procedures with longer surgical times—such as vitreoretinal surgeries—tend to have higher rates of macular edema and greater severity, particularly when the macula is affected by the primary disease or in the presence of vitreoretinal proliferation. The poorer outcomes associated with Ozurdex implantation at the time of silicone oil removal reinforce this hypothesis, as these eyes had previously undergone complex surgical procedures.

## Conclusion

Intravitreal Ozurdex implants significantly improved functional and structural aspects in post-surgical macular edema. The timing of implantation influenced VA improvement, with a distinct step approach showing better outcomes than at the time of oil removal.

## Data Availability

The data that support the findings of this study are available from the Hospital Ophthal Ltda but restrictions apply to the availability of these data, which were used under license for the current study, and so are not publicly available. Data are however available from the corresponding author upon reasonable request and with permission of Hospital Ophthal Ltda.
